# Application of silver nanofluid containing oleic acid surfactant in a thermosyphon economizer

**DOI:** 10.1186/1556-276X-6-315

**Published:** 2011-04-07

**Authors:** Thanya Parametthanuwat, Sampan Rittidech, Adisak Pattiya, Yulong Ding, Sanjeeva Witharana

**Affiliations:** 1 Heat-Pipe and Thermal Tools Design Research Unit (HTDR), Division of Mechanical Engineering, Faculty of Engineering, Mahasarakham University, Thailand; 2 Bio-Energy Research Laboratory (BERL), Division of Mechanical Engineering, Faculty of Engineering, Mahasarakham University, Thailand; 3 Institute of Particle Science & Engineering, University of Leeds, Leeds, UK

## Abstract

This article reports a recent study on the application of a two-phase closed thermosyphon (TPCT) in a thermosyphon for economizer (TPEC). The TPEC had three sections of equal size; the evaporator, the adiabatic section, and the condenser, of 250 mm × 250 mm × 250 mm (*W *× *L *× *H*). The TPCT was a steel tube of 12.7-mm ID. The filling ratios chosen to study were 30, 50, and 80% with respect to the evaporator length. The volumetric flow rates for the coolant (in the condenser) were 1, 2.5, and 5 l/min. Five working fluids investigated were: water, water-based silver nanofluid with silver concentration 0.5 w/v%, and the nanofluid (NF) mixed with 0.5, 1, and 1.5 w/v% of oleic acid (OA). The operating temperatures were 60, 70, and 80°C. Experimental data showed that the TPEC gave the highest heat flux of about 25 kW/m^2 ^and the highest effectiveness of about 0.3 at a filling ratio of 50%, with the nanofluid containing 1 w/v% of OA. It was further found that the effectiveness of nanofluid and the OA containing nanofluids were superior in effectiveness over water in all experimental conditions came under this study. Moreover, the presence of OA had clearly contributed to raise the effectiveness of the nanofluid.

## Introduction

**Figure 1:**
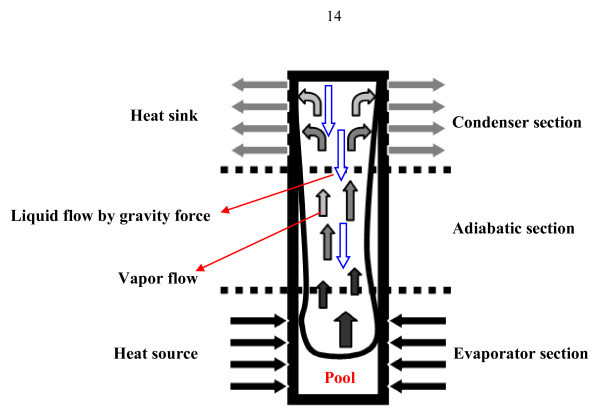
**Schematic of the two-phase closed thermosyphon**.

Two-phase closed thermosyphon (TPCT) as illustrated in Figure 1 is essentially a gravity-assisted wickless heat pipe, which utilizes the heat of evaporation and condensation of the working fluid. Contrary to the conventional heat pipe that uses the capillary force to return the liquid to evaporator, the TPCT uses gravity to return the condensate. Since the evaporator of a TPCT is located in the lowest position, the gravitational force will support the capillary force [1-3]. The TPCT has a number of advantages such as simple structure, very small thermal resistance, high efficiency, and low manufacturing costs. It has, therefore, been widely used in various applications such as in industrial heat recovery, electronic component cooling, turbine blade cooling, and solar heating systems [4-6]. The TPCT could be modified to suit many more applications such as heat exchangers and economizers. The first successful design of economizer was used to increase efficiency of boilers for stationary steam engines. It consisted of an array of vertical cast iron tubes connected to two tanks of water above and below, in-between which the exhaust gases from the boilers passed.

An economizer is a type of heat exchanger that can be classified into four types: tubular heat exchanger type (double pipe, shell and tube, and coil tube), plate heat exchanger type (gasketed, spiral, plate coil, and lamella), extended surface heat exchanger type (tube-fin and plate-fin), and regenerator type (fixed matrix and rotary) [7-9]. Nada et al. [10] used a TPCT in a solar collector with a shell and tube heat exchanger and observed a uniform temperature distribution [10]. The performance of a TPCT depends upon the aspect ratio (length to diameter) and the filling ratio (volume of fluid to volume of evaporator). Another application of the TPCT is in the energy recovery systems in air conditioning plants in tropical countries. There, the inlet air is pre-cooled by the cold exhaust stream before it enters the refrigeration equipment [11-13]. Lukitobudi et al. [14] studied the heat exchange from hot water to air using a TPCT, and Atipong et al. [15] studied oscillating heat pipe in a wire-on-tube heat exchanger. The results obtained by both groups showed that after the heat recovery, the effectiveness and heat transfer of the evaporator and condenser increased by about 48%. Mostafa et al. [16] reported that the economizer in the TPCT imposed limitation to the heat transfer due to the lower quality of the working fluid accumulated inside. When nanofluids were used as working fluids, they increased the thermal and heat transfer capacities. Nanofluids are created by suspending ultra-fine metallic or nonmetallic particles typically of several tens of nanometers in size, in base fluids such as water, oil, and ethylene glycol. Nanofluids were known to have enhanced the thermal conductivity and convective heat transfer. However, to obtain a sizable enhancement in thermal conductivity, the particle volume concentration needs to be significantly large, in the order of 0.5 vol% or above [17,18]. The distinct features of nanofluids are their stronger temperature-dependent thermal conductivity than the base fluid [19,20]. The thermal conductivity also depends upon the concentration of the added surfactant. In some instances, the nanofluids were unstable and the nanoparticles found to have precipitated. A surfactant improves the stability of a nanofluid by uniform dispersion of particles [21-23]. A surfactant can adsorb gas in a liquid-gas interface and decrease the interfacial tension. Some surfactants may flocculate in the bulk solution [24,25].

The TPEC used in this study was a special type that uses nanofluids in the thermosyphon to transfer heat from evaporator to condenser without external energy requirement. The primary objective of this study is to design and test the TPEC that will increase the heat transfer to water. The heat will be helpful to increase effectiveness of the TPEC. This TPEC was designed using a correlation of Kutateladza number (*Ku*).

### TPEC design, experimental apparatus, and analysis

#### TPEC design

An economizer kit was designed using the Kutateladza number (*Ku*) to predict the heat transfer of a TPCT. The TPEC had three sections of equal size; the evaporator, the adiabatic section, and the condenser, of 250 mm × 250 mm × 250 mm (*W *× *L *× *H*). The thermosyphon was made with steel tubes of 12.7-mm ID. The details of the economizer are shown in Table 1. Equation 1 was used to calculate the heat transfer rate of the system.(1)

**Table 1:** System design conditions

Section of economizer	Condition design
	Length was 250 mm
Evaporator section	Hot water flow was 80°C
	Volumetric flow rate was 5 l/min
Adiabatic section	Length was 250 mm
	Length was 250 mm
Condenser section	Cool water flow was 25°C
	Volumetric flow rate was 1 l/min

Then Equation 2 was used to calculate the convection heat transfer rate of system.(2)

Now it becomes:

The tube heat conduction loss was analyzed by Engineering Sciences Data Unit Data Item No. 80013 (ESDU 81038) method [8]. The wall heat conduction transfer rate loss was calculated using Fourier' law [26] as follows:(3)

Thus,

The aim of this research was to find a correlation to predict the heat transfer of the TPCT for a given number of tubes order to apply for TPEC. *Ku *is related to the aspects ratio () that represents the distance of physical motion for the working fluid (liquid and vapor). The dimensionless groups encountered are: *Prandtl number, Pr *(The ratio of momentum diffusivity to the thermal diffusivity of liquid. It represents convection heat transfer in a tube that occurs when the vapor bubble moves from the evaporator section to the condenser section.), *Bond number, Bo *(The ratio of buoyancy force to the surface tension force. *Bo *can be used to explain boiling phenomena inside the evaporator section and the state of vapor bubbles in nucleate boiling.), *Jacob number, Ja *(The ratio of latent heat to sensible heat of the working fluid. It represents the phase change of the working fluid). Note that if all the groups have values lower than 1; there will be no occurrence of phase change. *Peclet number, Pe*, is the ratio of bulk heat transfer rates to conductive heat transfer rates. *Condensation Number, Co*, is the liquid density ratio and hence the gravitational component and homogeneous theory for the momentum component (heat flux divided by the product of mass flux and latent heat of vaporization). The higher the value of *Co*, the easier for the condensate to return to the evaporator section. *Drag coefficient, Cd*, is proportional to gravitational to internal forces that predict momentum heat transfer rates dependent on the physical motion. *Archimedes number, Ar*, determines the motion of fluid and solids due to density differences. *Ar *is dependent on dimension to prediction the boiling phenomenon approaches boiling inside. *Ohnesorge number, Z*, is proportional to viscous force to inertial force with surface tension. *Z *is generally used in momentum heat transfer rates and atomization. The above-stated dimensionless numbers were correlated with *Ku *in the form of Equation 4 to calculate the convection heat transfer capacity of one tube.(4)

Thus,(5)

From Equations 4 and 5, the heat flux of the TPCT at a vertical position can be evaluated from the Equation 6:(6)

The calculations showed that the number of tubes for TPEC is 12.

#### Experimental apparatus

This section describes experimental setup, the parameters of the study, and the procedure. The experimental plan is given in Table 2.

**Table 2:** Controlled and variable parameters

	The tubes were arranged in a staggered
	Operating temperature of 60,70 and 80°C
The controlled parameters	Silver nanofluid concentration of 0.5 w/v%
	Volumetric flow rate was 5 l/min in evaporator section
	Cool water flow was 25°C in condenser section
	Working fluid = pure water, silver nanofluid concentration of 0.5 w/v% and silver nanofluid concentration of 0.5 w/v% mixed oleic acid surfactant
The variable parameters	Concentration of oleic acid surfactant were 0.5, 1, 1.5 w/v%
	Volumetric flow rate were 1, 2, 5 l/min in condenser section
	Filling ratio = 30, 50, and 80% (by total length of evaporator)

The nanofluid was produced by suspending metal or metal oxide nanoparticles in a base fluid such as water. The preparation involved several steps such as changing the pH value of the suspension, using surfactant activators, and using ultrasonic vibration. For this study, the nanofluid was sonicated for 5 h in ultrasonic bath. Silver nanopowder (<100 nm particle size, 99.9% metals basis) and oleic acid were obtained from Sigma-Aldrich Inc, Milwaukee, Wisconsin: USA. The silver nanoparticles were suspended in DI water with concentrations of 0.5 w/v% [16]. After that, the silver nanoparticles were suspended into de-ionized water with concentrations of 0.5 w/v% mixed with oleic acid surfactant concentration of 0.5, 1, and 1.5 w/v%, respectively. The nanofluids were stable for a long time.

**Figure 2:**
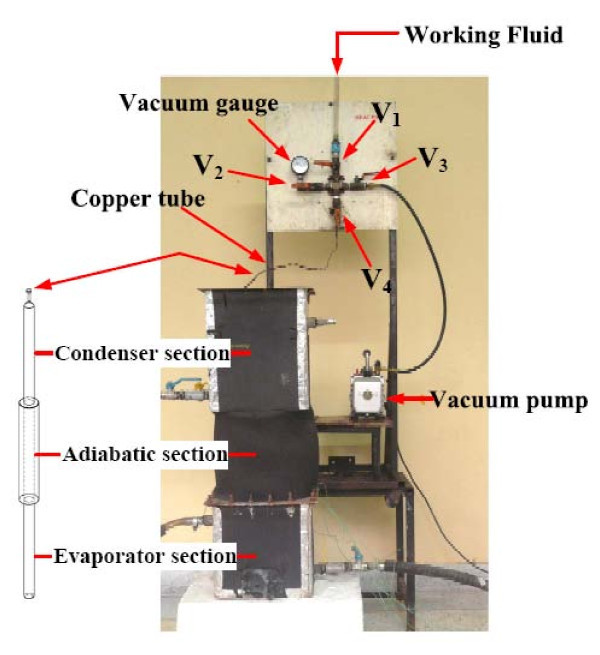
**Schematic of initially the TPCT is filling working fluid**.

The TPCT in economizer was 12 tubes by stand upright the copper tube over thermal from hot bath. After that, the TPCT were connected together with copper pipe. The copper pipe was breached to insert a valve mechanism that was used to evacuate and subsequently charge the TPCT with the working fluids. The charging procedure, as shown in Figure 2, consists of attaching a vacuum pump to the valve.

**Figure 3:**
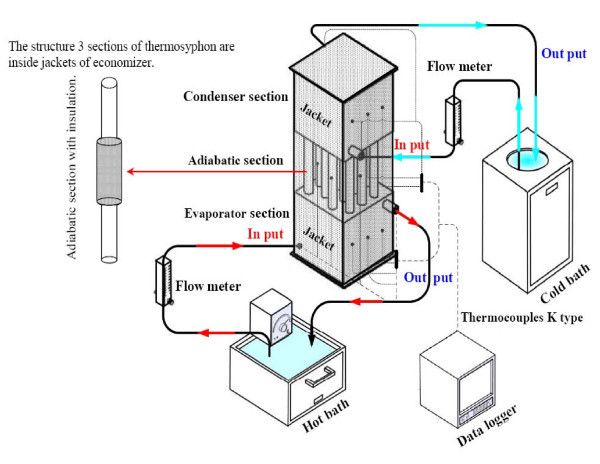
**Schematic diagram of experimental apparatus**.

Initially, the TPCT should be evacuated to about 0.010 mmHg. The time required to achieve this level depends on the pump capacity. Before filling the tube with the working fluid, the system was leak-checked with a vacuum gauge. This is done by closing valve V_1_, while leaving V_2_, V_3_, and V_4 _open. Then to fill the working fluid to the TPCT, open V_1 _and close V_3_. After the correct inventory of liquid was allowed into the TPCT, V_1 _was closed. Now valve V_3 _was opened and the vacuum pump was activated. While doing so the valve V_4 _was closed and the copper tube was dissected and a welding cap was placed on it. Now the TPCT was ready for experiment. Figure 3 shows the schematic diagram of the experimental apparatus which consists of a TPEC and peripheral devices. The evaporator section is the heat source with a hot bath. The condenser section is the heat sink with a cold bath. The heat was supplied by circulating water through the evaporator. The hot water flow rates were controlled to achieve ± 4°C temperature in the adiabatic section

The evaporator, the adiabatic, and the condenser sections of the TPEC were of equal aspect ratios. Thirteen thermocouples were connected through a data logger (Yokogawa DX200 with ±0.1°C accuracy, 20 channel input and -200 to 1100°C measurement temperature range). The type K thermocouples (OMEGA with ±0.1°C accuracy) were attached to the inlet and the outlet of the heating and cooling jackets as well as to the TPEC. Altogether there were five temperature measuring points on the condenser, five on the evaporator, and three on the adiabatic section. A hot bath (TECHNE TE-10D with an operating range of -40 to 120°C and ±0.1°C accuracy) was used to pump hot water into the heating jacket in the evaporator section and the cold bath (EYELA CA -1111, volume 6.0 l with an operating, temperature range of -20 to 30°C and ±2°C accuracy) was used to pump the cooling water into the cooling jacket in the condenser section. The inlet temperature of the cooling water was maintained at 20°C and a floating Rota meter (Blue point S-4-103 for a flow rate of 0.5-5 l/min) was used to measure the flow rate of water during the experiments. In order to calculate the heat transfer rate of the TPEC, Equation 2) was used. Equation 7 was subsequently used to determine the calculation error [16].(7)

#### The effectiveness analysis

To analyze the performance of the TPEC, the effectiveness (*ε*) was calculated by the Number of Transfer Unit Method (*ε *- NTU). The NTU is based on the heat exchanger effectiveness defined as the ratio of actual heat transfer in a heat exchanger to the maximum possible amount of heat that could be transferred with an infinite area [26].

**Figure 4:**
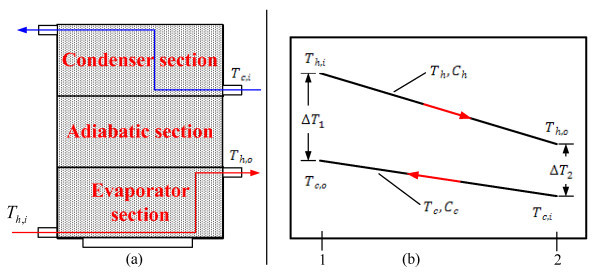
**(a) Flow diagram of experimental apparatus**. **(b) **Temperature distribution for a counter flow TPEC [27].

Figure 4a shows the fluid flow diagram and Figure 4b shows the typical temperature profiles for a counter-flow TPEC. For this scheme, the effectiveness can be written as [27]:(8)

where the minimum heat capacity is defined as:(9)

and the NTU is:(10)

Thus,

The effectiveness of a counter flow heat exchanger is:(11)

The experimental conditions are given in Table 2.

## Result and discussion

### Effect of operating temperature on heat flux

**Figure 5:**
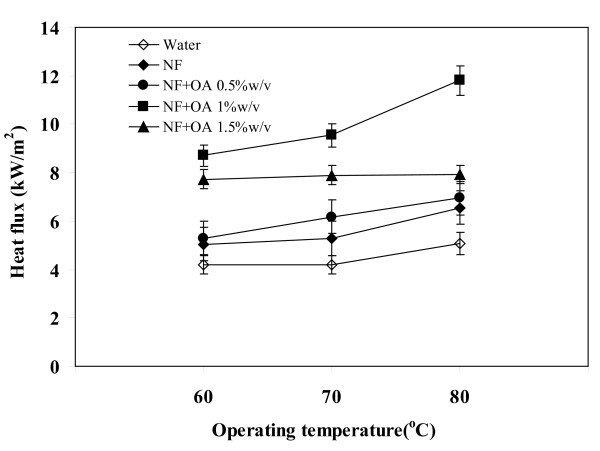
**Relationship between operating temperature and heat flux**. Volumetric flow rate = 1 l/min, filling ratio = 50%.

Dependence of the operating temperature on the heat flux of TPCE filled with the silver nanofluid mixed with oleic acid (NF + OA) is shown in Figure 5. Also shown are the data for water. In all cases the NF + OA shows superior performance than pure water. The maximum heat flux of 12 kW/m^2 ^has occurs with the OA 1 w/v% nanofluid at the operating temperature of 80°C. From this it can be seen that when the temperature was increased from 60 to 80°C, the heat flux had increased by different proportions. At this temperature interval, the pool boiling occurred that resulted high heat transfer rates. Nanoparticles present in the liquid can increase the surface area for heat absorption. As a consequence the liquid will raise its temperature quicker and start to boil. In the case of NF + OA, the OA will stabilize the nanoparticles by uniformly distributing them. This may cause increase in the thermal conductivity of the liquid, which in turn helps to raise the liquid temperature.

### Effect of filling ratios on heat flux

**Figure 6:**
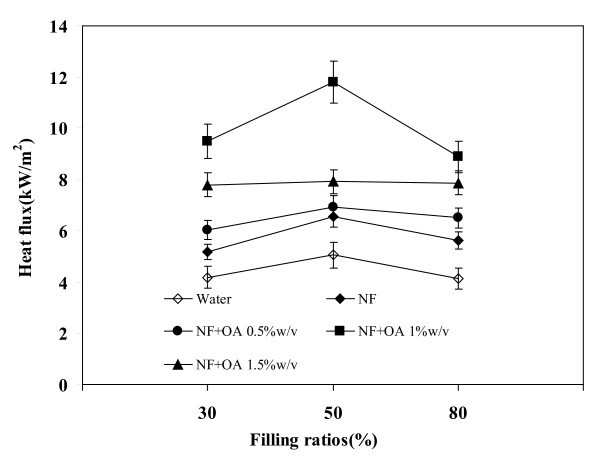
**Relationship between filling ratios and heat flux**. Volumetric flow rate = 1 l/min, operating temperature = 80°C.

Figure 6 shows the effect of filling ratios on heat flux. The maximum heat flux of 12 kW/m^2 ^has occurred at the filling ratio of 50% with the NF + OA 1 w/v%. This is approximately 60% higher than water. Filling ratios of 30 and 80% presumably caused dry out and flooding of the evaporator [1,5,13] which made the 50% filling ratio as the most favorable.

### Effect of volumetric flow rate on heat flux

**Figure 7:**
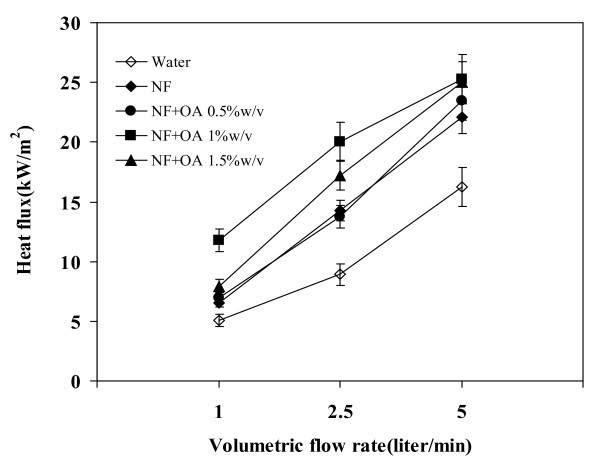
**Relationship between volumetric flow rate and heat flux**. Operating temperature = 80°C, filling ratio = 50%.

Relationship between the volumetric flow rate and the heat flux of TPEC at 80°C is shown in Figure 7. The heat flux has increased with the volumetric flow rate suggesting that the thermosyphon efficiency increasing with the same. Consider the case of 1 w/v% nanofluid, where at 5 l/min, the resulting heat flux was 25 kW/m^2^. The increase of the maximum heat flux with the volumetric flow rate can be attributed to the increase of the operating temperature. As the operating temperature increases, the system approaches boiling.

### Effect of concentration on effectiveness

**Figure 8:**
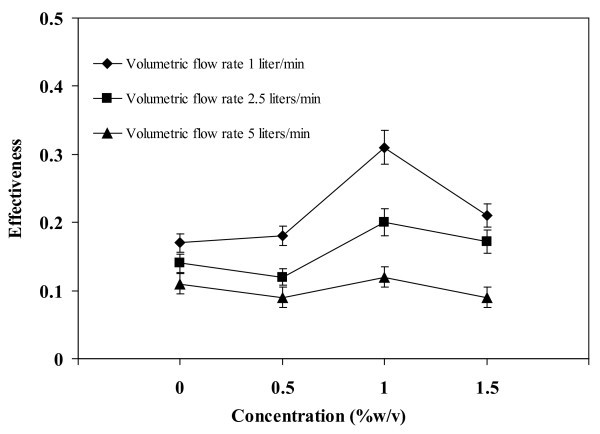
**Relationship between concentration (%w/v) and effectiveness**. Operating temperature = 80°C, filling ratio = 50%.

The experimental data for effectiveness versus the concentration of oleic acid surfactant in nanofluid are presented in Figure 8. The maximum effectiveness of 0.3 has occurred at OA concentration of 1 w/v%, which was better than OA concentrations of 0, 0.5, and 1.5 w/v%. This behavior could possibly be caused by the change in viscosity. When the OA concentration was smaller or larger than 1 w/v%, it was either insufficient to stabilize the nanofluid or introduced excessive oil to the surface that suppressed bubble movement. The possible influence of surface tension is explained in the following section.

### Effect of operating temperature on effectiveness

**Figure 9:**
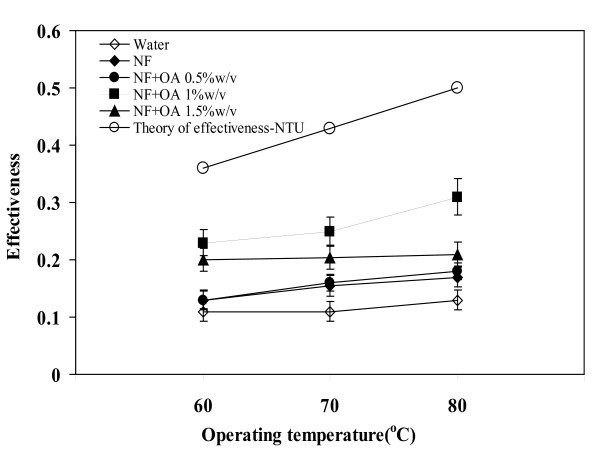
**Relationship between operating temperature and effectiveness**. Volumetric flow rate = 1 l/min, filling ratio = 50%.

The experimental data and theoretical predictions for the effect of operating temperature on the effectiveness of TPEC are demonstrated in Figure 9. The maximum effectiveness of 0.3 has occurred with the OA concentration of 1 w/v% and at 80°C. The effectiveness increased with the operating temperature. This is due to the onset of boiling in the TPCT and also due to the reduction of surface tension that made the bubbles easier to move upwards. In particular the addition of OA further reduced the surface tension that would cause early boiling. Figure 9 further shows that at 80°C, the effectiveness of water was 80% lower than the theory, whereas the effectiveness of NF + OA 1 w/v% was only 40% lower. Hence, the NF + OA has performed better than water. This demonstrates the benefit of NF + OA as a working fluid in TPCT.

### Effect of filling ratios on effectiveness

**Figure 10:**
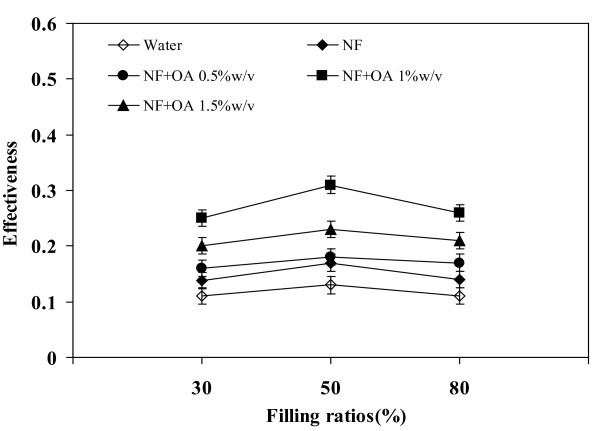
**Relationship between filling ratios and effectiveness**. Volumetric flow rate = 1 l/min, operating temperature = 80°C.

Figure 10 presents the experimental data for the effectiveness versus filling ratios. The maximum effectiveness of 0.3 has occurred at the filling ratio of 50% with the nanofluid mixed with OA 1 w/v%. The OA molecule has long chain length that helps to stabilize the nanofluid. From this data it suggests that 1 w/v% of OA is the optimal concentration.

### Effect of volumetric flow rate on effectiveness

**Figure 11:**
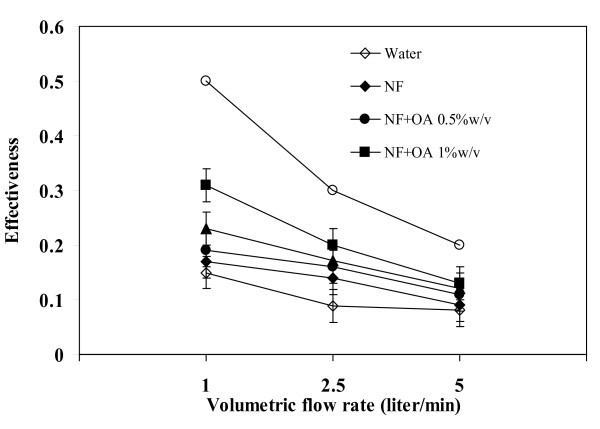
**Relationship between volumetric flow rate and effectiveness**. Operating temperature = 80°C, filling ratio = 50%.

It can be seen from Figure 11 that the effectiveness of TPEC has strong dependence on the volumetric flow rate. The maximum effectiveness obtained from experiments was 0.3 that occurred at 1 l/min, for which the theoretical prediction was 0.5. When the flow rate was increased, the amount of water in the condenser also increased that caused the reduction of the effectiveness. This observation agrees with Equation 8.

## Conclusions

A TPEC was designed using a correlation of Kutateladza number (*Ku*) for the prediction of heat transfer of the TPCT. Experiments were conducted on the TPEC using various working fluids to study the effects of various parameters on the heat flux and the effectiveness. It was found that pure water gave the lowest values for heat flux, whereas the silver nanofluid and the silver nanofluid containing oleic acid gave the higher heat fluxes. In particular, the silver nanofluid containing 1 w/v% oleic acid exhibited the best performance in all experiments. Moreover 80°C operating temperature, 50% filling ratio, 5 l/min volumetric flow rate were proved to be the optimum working conditions that yielded the maximum heat flux from this TPEC. Furthermore, it was found that the highest value for effectiveness was also displayed by the silver nanofluid containing 1 w/v% oleic acid at 80°C operating temperature, 50% filling ratio, and 1 l/min volumetric flow rate.

### List of symbols

*A *Total heat transfer area, surface area of evaporator (m^2^)

*C *Capacity rate (kJ(s°C)^-1^)

*C*_*p *_Specific heat capacity constant pressure, (J(kg °C)^-1^)

*D *Diameter (m)

*h*_*fg *_Latent heat of vaporization, (kJ · kg^-1^)

*k *Thermal conductivity (W/mK)

*L *Length of thermosyphon (mm)

*L*_c _Characteristic length (m)

 Mass flow rate (kg · s^-1^)

NF Silver nanofluid

NF + OA Silver nanofluid with oleic acid

NF + OA 0.5 w/v% Silver nanofluid with oleic acid concentration 0.5 w/v%

NF + OA 1 w/v% Silver nanofluid with oleic acid concentration 1 w/v%

NF + OA 1.5 w/v% Silver nanofluid with oleic acid concentration 1.5 w/v%

OA Oleic acid

*Q *Heat transfer rate (W)

*q *Heat flux (kW/m^2^)

*T*_out _Outlet temperature at condenser section (°C)

*T*_in _Inlet temperature at condenser section (°C)

*T*_*v *_Operating temperature (°C)

Δ*T *Temperature difference (°C)

*U *Overall heat transfer coefficient (W · m^-2 ^· K)

*V *Velocity (m · s^-1^)

### Greek symbols

ρ Density (kg · m^-3^)

μ Viscosity (Pa · s)

σ Surface tension (N · m^-1^)

*ε *Effectiveness of economizer

### Subscripts

a Adiabatic

c Condenser, cold fluid

e Evaporator

h Hot fluid

i in

l Liquid

max Maximum

min Minimum

o out

v Vapor

*Ar*, Archimedes number = 

*Bo*, Bond number = 

*Co*, Condensation number = 

*Ja*, Jacob number = 

*Ku*, Kutateladza number = 

Aspect ratio = 

*Pr*, Prandtl number = 

*Pe*, Peclet number = 

*Cd*, Drag number = 

*Z*, Ohensorge number = 

## Abbreviations

NF: nanofluid; OA: oleic acid; TPEC: thermosyphon for economizer; TPCT: two-phase closed thermosyphon.

## Competing interests

The authors declare that they have no competing interests.

## Authors' contributions

TP conducted the experiments. SR helped and supervised TP for experiments. AP and YD supervised and facilitated the work in their respective institutions. SW revised and edited the manuscript. All authors read and approved the final manuscript.
